# Epidemiology of 3 Vaccine-Preventable Infectious Diseases Within US Immigration Detention Centers

**DOI:** 10.1001/jamanetworkopen.2025.44278

**Published:** 2025-10-22

**Authors:** Ribhav Gupta, Dean Winslow, Ronit Gupta, Sten H. Vermund

**Affiliations:** 1 Department of Medicine, University of Minnesota School of Medicine, Minneapolis; 2 Department of Epidemiology of Microbial Disease, Yale School of Public Health, New Haven, Connecticut; 3 Department of Emergency Medicine, Stanford University School of Medicine, Stanford, California; 4 Department of Medicine, Stanford University School of Medicine, Stanford, California; 5 Department of Pediatrics, Stanford University School of Medicine, Stanford, California; 6 Center for International Security and Cooperation, Stanford University, Stanford, California; 7 Center for Disease Control and Prevention, Atlanta, Georgia; 8 Department of Biostatistics, Harvard T.H. Chan School of Public Health, Boston, Massachusetts; 9 Department of Medicine, Dartmouth Geisel School of Medicine, Hanover, New Hampshire; 10 Office of the Dean, University of South Florida College of Public Health, Tampa; 11 The Global Virus Network, Tampa, Florida

## Abstract

**Importance:**

Migrants detained by US Immigration and Customs Enforcement (ICE) are at risk of preventable infections from crowding and limited health care. Postpandemic risk remains unclear as detention increases.

**Objective:**

To assess epidemiological patterns of 3 vaccine-preventable diseases within ICE facilities.

**Design, Setting, and Participants:**

This case series study examined data from January 2019 through October 2023 from ICE facilities managed by ICE Health Services Corp during the study period. All cases of influenza, mumps, and hepatitis A among persons detained in reporting ICE facilities were included.

**Exposure:**

Diagnosis of influenza, mumps, or hepatitis A during ICE detention.

**Main Outcomes and Measures:**

The primary outcomes were monthly case counts and estimated case rates (per 100 000 person-months), outbreak occurrence and duration, and seasonal and geospatial patterns. Outbreaks were defined as 3 or more cases within a facility over at least 1 month. Seasonal variation was assessed through variations in mean monthly case rates across years with analysis of variance, and Tukey tests were used to compare monthly pairwise permutations for statistical significance. Geospatial trends were evaluated using global and local Moran *I* analyses of annual facility-level case rates.

**Results:**

From 2019 to 2023, at a total of 20 ICE facilities, there were 2035 influenza cases (mean [SD] age, 33.1 [10.8] years; 1804 [88.6%] men), with a mean of 35.1 cases monthly (range, 0.0-276.0 cases monthly). The mean facility-level monthly case rate was 17.3 cases per 100 000 persons (range, 0.0-720.0 cases per 100 000 persons), lower than national rates. December had the highest case rate (mean [SD], 59.4 [142.0] cases per 100 000 persons) and September the lowest (mean [SD], 6.1 [11.9] cases per 100 000 persons), paralleling national trends. Across 15 facilities, 79 outbreaks occurred involving 1739 cases with a mean duration of 2.5 months (range, 1.0-13.0 months). For mumps, 252 cases were reported (mean [SD] age, 34.3 [9.2] years; 235 [93.3%] men), with a mean of 4.3 cases monthly (range, 0.0-61.0 cases monthly). The mean facility-level monthly case rate was 1.5 cases per 100 000 persons (range, 0.0-160.2 cases per 100 000 persons). June had the highest mean case rate (3.1 cases per 100 000 persons) and February the lowest (0.1 cases per 100 000 persons); 16 outbreaks across 8 facilities involved 177 cases with a mean duration of 1.8 months (range, 1.0-6.0 months). For hepatitis A, there were 486 reported cases (mean [SD] age, 39.2 [10.8] years; 408 [84.0%] men), with a mean of 8.4 cases monthly (range, 0.0-40.0 cases monthly). The mean facility-level monthly case rate was 6.0 cases per 100 000 persons (range, 0.0-273.4 cases per 100 000 persons). July had the highest mean case rate (13.8 cases) and November the lowest (2.3 cases); 33 outbreaks across 11 facilities involved 158 cases with a mean duration of 1.2 months (range, 1.0-2.0 months). No geospatial clusters were found.

**Conclusions and Relevance:**

This case series found that vaccine-preventable infectious disease burden among individuals detained by ICE varied by season and facility. Mitigation could include decongregation, reduced crowding, vaccinations, and improved infection control procedures to protect migrants and staff.

## Key Points

**Question:**

What were the burden and transmission patterns of influenza, mumps, and hepatitis A in US Immigration and Customs Enforcement detention facilities from 2019 to 2023, and what risks did they pose to detained migrants and staff?

**Findings:**

This case series of 20 facilities found that influenza (2035 cases), mumps (252 cases), and hepatitis A (486 cases) were all prevalent with varying seasonality and multiple facility outbreaks, reflecting the risk profiles for respiratory and enteric infectious diseases of persons entering detention.

**Meaning:**

On the basis of these findings, decongregation, control of facility crowding, and vaccination programs are recommended approaches to reduce transmission risks for incarcerated migrants and facility employees.

## Teaser

This case series examines epidemiological patterns of 3 vaccine-preventable diseases within US Immigration and Customs Enforcement facilities.

## Introduction

Detained migrants living in US Immigration and Customs Enforcement (ICE) agency detention facilities are reported to be at higher risk of contracting infectious diseases.^1,2,3,4,5,6,7^ This risk is particularly pronounced for vaccine-preventable diseases, including influenza, COVID-19, and measles.^1,3,4,7^ High COVID-19 case rates prompted a House Committee hearing on ICE’s prevaccine response.^8^ Suboptimal immunization rates in this population are compounded by limited baseline vaccination data; multiple outbreaks (notably for measles) and preventable infection-related deaths have been reported, including of children.^3,7^ Contributing factors include crowding, suboptimal sanitation, limited health care access, poor ventilation, and inconsistent diagnostic practices.^2,3,5,9^

Health care within ICE facilities is delivered by health department staff, contractors, and/or the ICE Health Service Corps (IHSC). The IHSC, with 1750 practitioners (as of 2024), oversees this system with the aim to deliver “high-quality health care to noncitizens in ICE custody.”^10,11^

The 147 facilities composing the ICE detention system in 2024 spanned approximately 47 US states and territories, with centers clustered in Texas, California, Florida, New York, and Arizona.^12,13^ These facilities included ICE-operated centers, private facilities, local jails, federal prisons, and field offices, with 86% of individuals historically held in for-profit facilities.^14^ The largest proportions of people detained were in Texas, California, Arizona, Louisiana, and New Mexico; most individuals experienced at least 1 interfacility transfer.^14,15^

In 2024, the IHSC directly staffed 18 of 147 detention facilities while overseeing care for over 300 000 persons, up from 200 000 people in 2022, with incremental budget increases lagging behind demand shifts.^10,11,16^ IHSC’s obligations are outlined in the US National Detention Standards, updated in 2019. These include an intake examination (medical, mental health, and dental), emergency care, follow-ups, and specialty services as needed on the basis of clinical discretion.^17^ Except for COVID-19, there are no standards for vaccinations.^17,18^ Among infectious diseases, only tuberculosis screening is mandated with additional testing left to clinician discretion.^17^

Although infection surveillance data remain limited, ICE has published COVID-19 case data, with organizations, including the Vera Institute, archiving records.^19^ Previous efforts have examined the epidemiology of select infectious diseases, most in the pre–COVID-19 period before the latest National Detention Standards. These studies have been limited in scope, duration, and infections covered.^1,3,7,20^ We analyzed 5-year trends (2019-2023) for 2 respiratory (influenza and mumps) and 1 enteric (hepatitis A) viruses within ICE facilities.

## Methods

### Data Source and Processing

This case study used deidentified data and was exempted from review and the need for informed consent per the University of Minnesota institutional review board. This study follows the reporting guideline for case series. Deidentified, individual-level data were provided by the Department of Homeland Security through the Freedom of Information Act for 8 communicable diseases over 1 decade (filed October 2023) from available facilities. As defined by *International Classification of Diseases, Ninth Revision* codes, we assessed hepatitis A, HIV, influenza, measles, mumps, tetanus, tuberculosis, and typhoid case data from November 2013 to October 2023. We focused on the 6 acute vaccine-preventable diseases, excluding HIV and tuberculosis, which are explored separately. Data were provided for facilities managed, at any point during this period, by IHSC. No data were provided for facilities not directly managed by IHSC (127 of 147 facilities in 2024). Additional data included age, sex, and country of origin. To preserve confidentiality and ensure analytical power, we excluded diseases (measles, typhoid, and tetanus) with fewer than 50 cases. We collected weekly national influenza hospital visit counts from the Centers for Disease Control and Prevention FluView database.^21^

We obtained annualized, facility-level mean daily populations from annual reports for facilities with case data.^22,23,24,25,26,27^ Available population data were not stratified. On the basis of temporal overlaps of case and population datasets, the primary analyses covered January 2019 through October 2023. Supplemental analyses included case count data from November 2013 through October 2023 (eFigures 1 and 2 in Supplement 1).

### Statistical Analysis

 Analyses were performed using R statistical software version 4.2.0 (R Project for Statistical Computing); analytic files are available online.^28^ We performed descriptive analyses of case demographics at the system and facility levels from 2019 to 2023. This included mean age, sex, and countries of origin with the highest case counts (ie, representing >1% of cases). System-level analyses included all IHSC-managed ICE facilities with available case data.

Monthly facility-level case rates from January 2019 to October 2023 were estimated using monthly case counts and annualized mean daily populations where available. Case rates were reported per 100 000 person-months to ease data interpretation. Person-months were measured as the mean daily population multiplied by days per month. The national influenza case rate was estimated using monthly case counts and annual mid-year census-based population projections.^29,30^ We mapped trends and estimated case rates at system and facility levels. For influenza, we mapped system-level and facility-level trends against national trends.

We examined seasonal variation in case rates. We compared facility-level case rates across months with a 1-way analysis of variance (ANOVA) test and, where significant, compared monthly pairwise permutations using the Tukey honest significant difference (HSD) test. To study case clusters, we performed an outbreak analysis based on a definition from prior literature, defining an outbreak as 3 or more cases diagnosed within the same facility during the same discrete month, and estimated outbreak duration and case intensity (ie, number of outbreak cases).^1^ Facilities were paired with corresponding zip codes in a geospatial analysis using global and local Moran *I* tests of the case rate annually and over the study period. Coordinates were determined from zip code centroids. We calculated spatial weights for each facility from their distance to the 5 most proximal facilities. Local Moran *I* statistics were considered significant (ie, indicative of a spatial hot spot) if the index value was greater than 0 and *P* < .10 (a lower threshold was selected given the exploratory study design and study population vulnerability). Additional details are shown in eAppendix 1 in Supplement 1.

## Results

### Overview

From January 2019 to September 2023, 2035 influenza cases, 486 hepatitis A cases, 252 mumps cases, and 1 case of each measles, typhoid, and tetanus were reported across the 20 IHSC-managed facilities with available data. Monthly facility-level case rates were obtained from 18 of 20 facilities. Men accounted for 79.8% (427.3 of 535.1 people) of the mean daily population across reporting facilities over the study period. Additional demographics are reported in the eTable in Supplement 1.

### Influenza

The mean (SD) age of persons with influenza was 33.1 (10.8) years, ranging across facilities from a mean (SD) of 21.8 (12.5) to 63.7 (0.0) years (Table). Men accounted for 1804 influenza cases (88.6%), ranging from 31.1% (50 men) to 100.0% (13 men) by facility and month. Influenza cases were primarily diagnosed among migrants from Honduras (235 individuals [13.0%]), Nicaragua (258 individuals [12.7%]), Mexico (252 individuals [12.4%]), Guatemala (234 individuals [11.5%]), Colombia (199 individuals [9.8%]), El Salvador (103 individuals [5.1%]), Ecuador (89 individuals [4.4%]), Peru (89 individuals [4.4%]), Cuba (86 individuals [4.2%]), Venezuela (76 individuals [3.7%]), Brazil (52 individuals [2.6%]), Turkey (48 individuals [2.4%]), Dominican Republic (38 individuals [1.9%]), and India (33 individuals [1.6%]) (Table).

**Table.** Demographic Characteristics of Reported Influenza, Mumps, and Hepatitis A Cases Among Detained Individuals

Characteristic	Individuals, No. (%)		
	Influenza (n = 2035)	Mumps (n = 252)	Hepatitis A (n = 486)
Demographics			
Age, mean (SD), y			
System level	33.1 (10.8)	34.3 (9.2)	39.2 (10.8)
Facility level range	21.8 (12.5) to 63.7 (0)^a^	30.4 (5.2) to 42.2 (14.5)	30.2 (9.4) to 52.4 (0)^a^
Sex			
Male			
System level	1804 (88.6)	235 (93.3)	408 (84.0)
Facility level range	1 (100) to 343 (99.4)	0 (0) to 54 (100)^a^	1 (50) to 98 (100)
Female			
System level	231 (11.4)	17 (6.8)	78 (16.0)
Facility level range	0 (0) to 111 (68.9)^a^	0 (0) to 5 (100)^a^	0 (0) to 39 (52.4)^a^
Country of origin^b^			
Bangladesh	0	7 (2.8)	2 (0.4)
Brazil	52 (2.6)	2 (0.8)	9 (1.9)
Cambodia	2 (0.1)	3 (1.2)	0
Cameroon	3 (0.1)	0	6 (1.2)
China	20 (1.0)	0	8 (1.6)
Colombia	199 (9.8)	0	34 (7.0)
Cuba	86 (4.2)	33 (13.1)	24 (4.9)
Democratic Republic of the Congo	4 (0.2)	4 (1.6)	1 (0.2)
Dominican Republic	38 (1.9)	0	17 (3.5)
Ecuador	89 (4.4)	18 (7.1)	19 (3.9)
El Salvador	103 (5.1)	22 (8.7)	36 (7.4)
Guatemala	234 (11.5)	33 (13.1)	49 (10.1)
Haiti	10 (0.5)	0	18 (3.7)
Honduras	265 (13.0)	71 (28.2)	54 (11.1)
India	33 (1.6)	0	5 (1.0)
Jamaica	1 (<0.1)	3 (1.2)	6 (1.2)
Mexico	252 (12.4)	20 (7.9)	43 (8.8)
Nicaragua	258 (12.7)	17 (6.7)	24 (4.9)
Peru	89 (4.4)	0	25 (5.1)
Senegal	7 (0.3)	3 (1.2)	11 (2.3)
Sierra Leone	0	0	7 (1.4)
Turkey	48 (2.4)	0	3 (0.6)
Uganda	0	4 (1.6)	0
Venezuela	76 (3.7)	1 (0.4)	22 (4.5)
Other	166 (8.2)	11 (4.4)	63 (13.0)

^a^ Values of zero indicate that there were too few cases in the facility to calculate an SD.

^b^ Only countries of origin from which more than 1% of all cases for any infection are included in the table.

**Figure 1.**
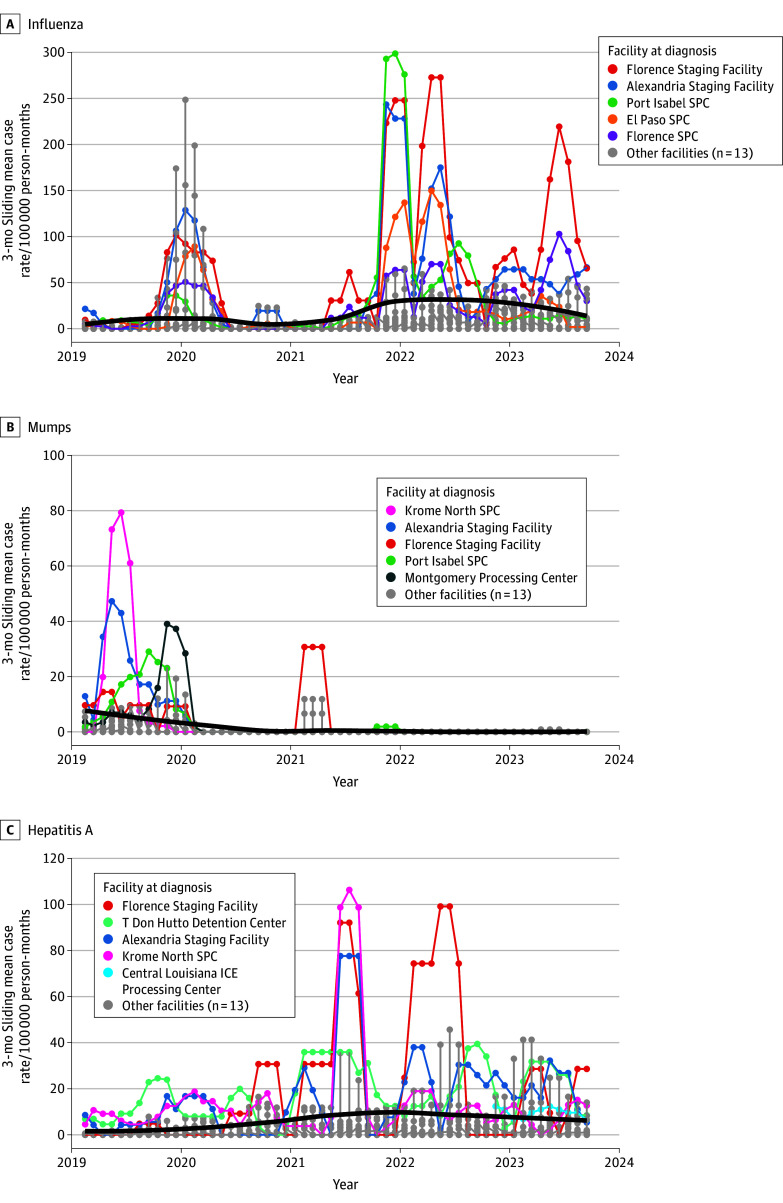
Disease Case Rates Over Time, by Reporting Detention Facility Graphs show 3-month sliding mean case rates (per 100 000 person-months) for influenza (A), mumps (B), and hepatitis A (C) at the 5 facilities with the highest disease-specific case rates from 2019 through 2023. Black line is the mean, unweighted facility-level case rate with a shadow of the SD. Variation in y-axis scale is dependent on disease panel. ICE indicates Immigration and Customs Enforcement; SPC, Service Processing Center.

From 2019 to 2023, the mean facility-level monthly influenza case rate was 17.3 cases per 100 000 person-months, varying from 0 (in multiple months and facilities) to 720.0 cases per 100 000 person-months (Port Isabel Service Processing Center [SPC] in December 2021) (Figure 1A; eFigures 3 and 4 in Supplement 1). Excluding an increase in early 2020, the case rate remained stable from 2019 to 2021, peaked in early 2022, and declined thereafter. Systemwide influenza cases counts increased over time with a monthly mean of 35.1 cases, ranging from 0 (multiple months) to 276 cases (December 2021) (eFigure 5 in Supplement 1). At the facility level, the mean monthly influenza case count was 1.8 cases, varying across facilities from fewer than 0.1 (NYC Holding Room) to 6.0 cases (Port Isabel SPC) (eFigure 6 in Supplement 1).

**Figure 2.**
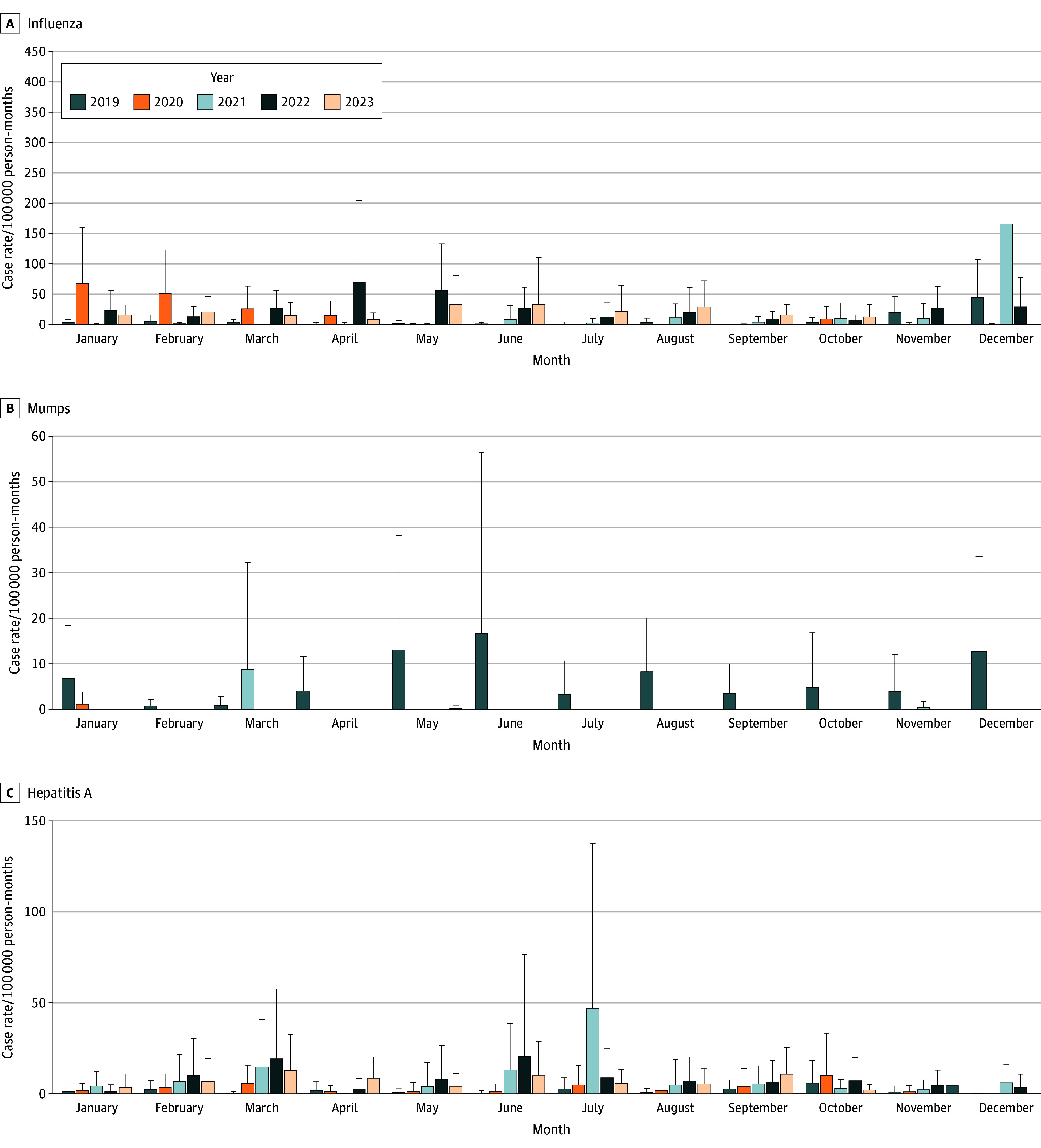
Seasonal Analysis of Variations in Facility-Level Case Rate by Month Bar plots show mean facility-level case rate per year and grouped by month for influenza (A),mumps (B), and hepatitis A (C) for 2019 to 2023. Variation in y-axis scale is dependent on disease panel. Error bars denote 95% CIs.

Seasonal analyses showed that the month with the highest mean influenza case rate was December (mean [SD], 59.4 [142.0] cases per 100 000 person-months), with the lowest case rate in September (mean [SD], 6.1 [11.9] cases per 100 000 person-months) (Figure 2A). A 1-way ANOVA test demonstrated significant differences in influenza case rate across months (*F*_11,977_ = 5.16; *P* < .001), with a Tukey HSD test demonstrating the case rate in December to be significantly greater than those in other months.

**Figure 3.**
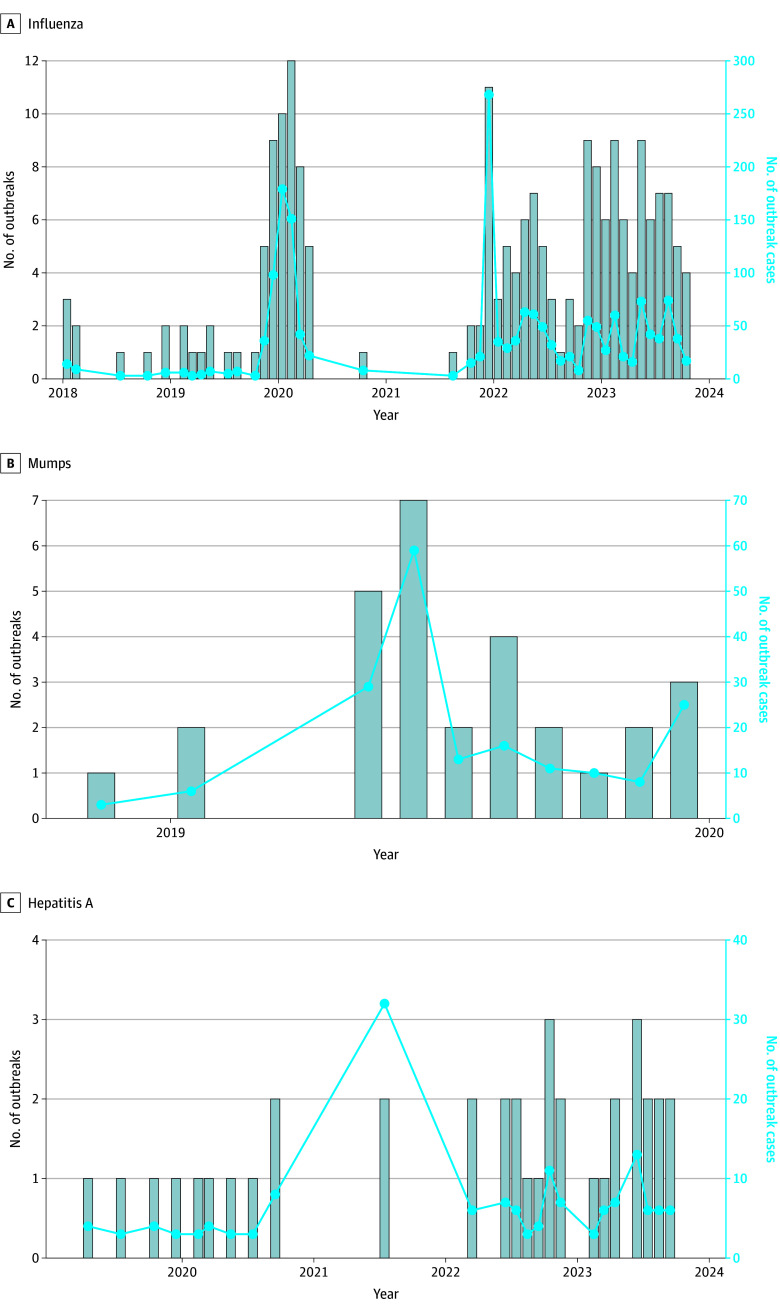
Number of Outbreaks and Outbreak Cases Over Time Across the System Bar plots show number of facilities with active outbreaks per month, and line plots show number of system-level outbreak cases over time for influenza (A), mumps (B), and hepatitis A (C) from 2019 to 2023. Variation in x-axis and y-axis scale is dependent on disease panel.

Fifteen facilities reported data compatible with 79 influenza outbreaks, accounting for 61.7% of 128 total outbreaks identified across al 3 diseases (Figure 3A). From February 2019 to October 2023, 1739 of 2035 possible outbreak-related cases (85.5%) were diagnosed. The mean duration of an influenza outbreak was 2.5 months, ranging from 1 month (multiple outbreaks) to 13 months (September 2022 to September 2023 in South Texas ICE Processing Center). The mean influenza outbreak case count was 22 cases, ranging from 3 cases (multiple outbreaks) to 185 cases (Port Isabel SPC from October 2021 to March 2022). The number of outbreaks per facility followed a normal distribution, ranging from 1 outbreak (multiple facilities) to 13 outbreaks (Port Isabel SPC) per facility.

A comparison of national-level and facility-level influenza case rates found similar trends over time (eFigures 7 and 8 in Supplement 1). National influenza rates broadly mirrored those across ICE facilities and were generally higher, although some facilities reporting higher case rates during seasonal peaks when compared to national estimates (eFigure 8 in Supplement 1).

**Figure 4.**
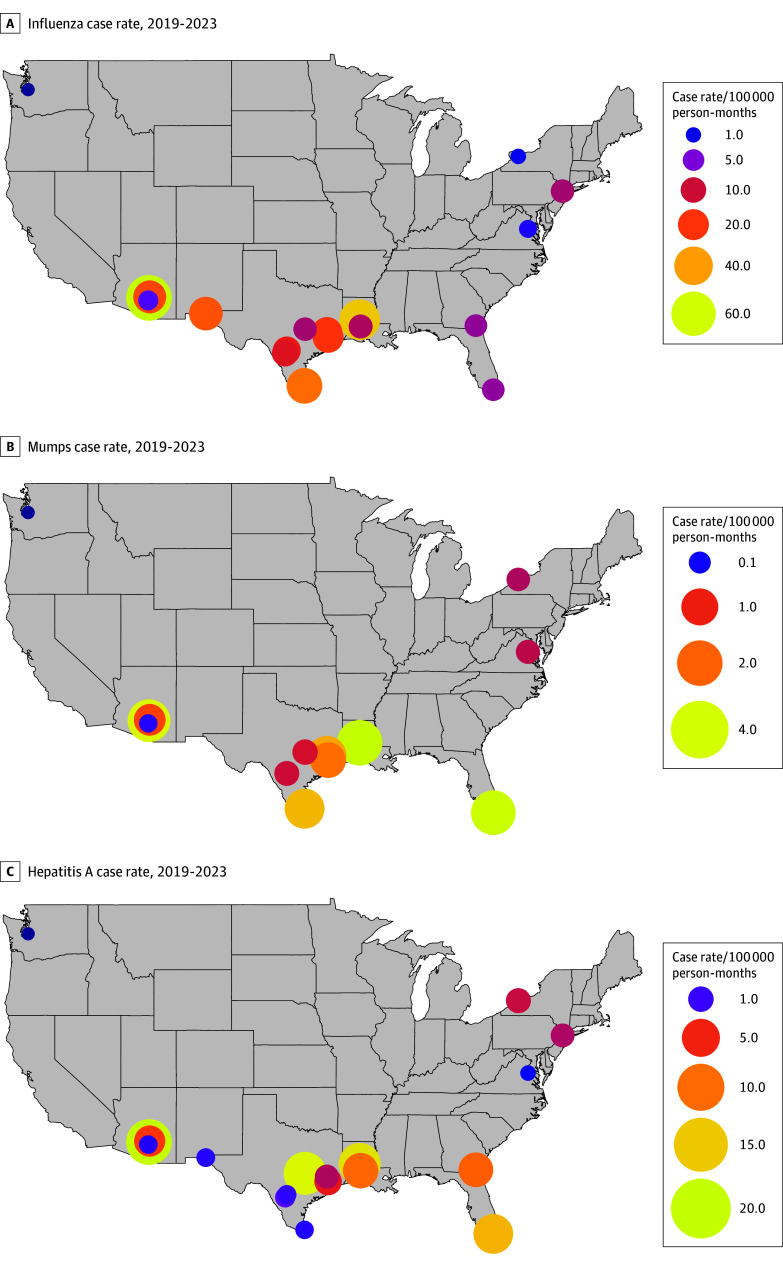
Spatial Distribution of Mean Case Rate at Facility Level Choropleth maps show mean case rate (per 100 000 person-months) at facility level for influenza (A), mumps (B), and hepatitis A (C) from 2019 to 2023. Circle size and color correspond to case rate. Please note variations in legends across panels.

Influenza geospatial clustering analyses visually suggested higher case rates in Texas and Arizona facilities, although no spatial clusters were confirmed (global Moran *I* = −0.02; *P* = .49) (Figure 4A). Local Moran *I* analyses demonstrated no regional influenza hot spots (or case clusters) over the study period or within each calendar year.

### Mumps

The mean (SD) age of persons with mumps was 34.3 (9.2) years, ranging across facilities from a mean (SD) of 30.4 (5.2) to 42.2 (14.5) years (Table). Men accounted for 235 mumps cases (93.3%), ranging from 0% to 100% (54 men) across facilities and months. Sixty-two mumps cases (24.6%) involved known complications, including orchitis. Mumps cases were primarily diagnosed among migrants from Honduras (71 individuals [28.2%]), Cuba (33 individuals [13.1%]), Guatemala (33 individuals [13.1%]), El Salvador (22 individuals [8.7%]), Mexico (20 individuals [7.9%]), Ecuador (18 individuals [7.1%]), Nicaragua (17 individuals [6.7%]), Bangladesh (7 individuals [2.8%]), Democratic Republic of the Congo (4 individuals [1.6%]), Uganda (4 individuals [1.6%]), Cambodia (3 individuals [1.2%]), Jamaica (3 individuals [1.2%]), and Senegal (3 individuals [1.2%]) (Table).

From 2019 to 2023, the mean facility-level monthly mumps case rate was 1.5 cases per 100 000 person-months, varying from 0 (multiple months and facilities) to 160.2 cases per 100 000 person-months (Krome North SPC in June 2019) (Figure 1B; eFigures 3 and 4 in Supplement 1). Following a peak in late 2019, the case rate has declined, except for a transient increase in early 2021. Although rates declined, systemwide mumps cases counts increased over time (as did the number of migrants detained) with a mean of 4.3 cases monthly, ranging from 0 (multiple months) to 61 cases monthly (June 2019) (eFigure 5 in Supplement 1). At the facility level, the mean monthly mumps case count was 0.2 cases, varying across facilities from 0 (multiple facilities) to 0.9 cases (multiple facilities) (eFigure 6 in Supplement 1).

Seasonal analyses showed that the month with the highest mean mumps case rate was June (mean [SD], 3.1 [18.0] cases per 100 000 person-months), with the lowest case rate in February (mean [SD], 0.1 [0.6] cases per 100 000 person-months) (Figure 2B). A 1-way ANOVA test demonstrated no significant differences in mumps case rates across months (*F*_11,977_ = 3.03; *P* = .35).

We identified 16 mumps outbreaks (Figure 3B) across 8 of 20 facilities (40.0%); 177 of 252 possible outbreak-related cases (70.2%) were diagnosed from January 2019 to December 2019. The mean duration of a mumps outbreak was 1.8 months, ranging from 1 month (multiple outbreaks) to 6 months (May 2019 to October 2019 in Port Isabel SPC). The mean mumps outbreak case count was 11.1 cases, ranging from 3 cases (multiple outbreaks) to 52 cases (Krome North SPC from May 2019 to July 2019). The number of outbreaks per facility followed a normal distribution, ranging from 1 outbreak (multiple facilities) to 3 outbreaks (multiple facilities) per facility.

Mumps geospatial clustering analyses visually suggested case rates were highest across facilities in Texas, Louisiana, and Florida, although no clusters were confirmed (global Moran *I* = −0.51; *P* = .69) (Figure 4B). Local Moran *I* analyses demonstrated no regional mumps hot spots over the study period or within each calendar year.

### Hepatitis A

The mean (SD) age of persons with hepatitis A was 39.2 (10.8) years, ranging across facilities from a mean (SD) of 30.2 (9.4) to 52.4 (0.0) years (Table). Men accounted for 408 hepatitis A cases (84.0%), ranging from 50% (1 man) to 100% (98 men) of cases across facilities. No cases of hepatic coma were recorded. Hepatitis A cases were primarily diagnosed among migrants from Honduras (54 individuals [11.1%]), Guatemala (49 individuals [10.1%]), Mexico (43 individuals [8.8%]), El Salvador (36 individuals [7.4%]), Colombia (34 individuals [7.0%]), Peru (25 individuals [5.1%]), Cuba (24 individuals [4.9%]), Nicaragua (24 individuals [4.9%]), Venezuela (22 individuals [4.5%]), Ecuador (19 individuals [3.9%]), Haiti (18 individuals [3.7%]), Dominican Republic (17 individuals [3.5%]), Senegal (11 individuals [2.3%]), Brazil (9 individuals [1.9%]), China (8 individuals [1.6%]), Sierra Leone (7 individuals [1.4%]), Cameroon (6 individuals [1.2%]), Jamaica (6 individuals [1.2%]), and India (5 individuals [1.0%]) (Table).

From 2019 to 2023, the mean facility-level monthly hepatitis A case rate was 6.0 cases per 100 000 person-months, varying from 0 (multiple months and facilities) to 273.4 cases per 100 000 person-months (Krome North SPC in July 2021) (Figure 1C; eFigures 3 and 4 in Supplement 1). From 2019 through mid-2021, the case rate increased before subsequently plateauing. Systemwide, the hepatitis A case count increased over time with a mean of 8.4 cases monthly, ranging from 0 (multiple months) to 40 cases monthly (July 2021) (eFigure 5 in Supplement 1). At the facility level, the mean monthly hepatitis A case count was 0.4 cases and varied across facilities from less than 0.1 case (multiple facilities) to 1.7 cases (Krome North SPC) (eFigure 6 in Supplement 1).

Seasonal analyses showed that the month with the highest mean hepatitis A case rate was July (mean [SD], 13.8 [43.9] cases per 100 000 person-months), with the lowest case rate in November (mean [SD], 2.3 [5.6] cases per 100 000 person-months) (Figure 2C). A 1-way ANOVA test demonstrated significant differences in hepatitis A case rates across months (*F*_11,977_ = 1.11; *P* < .001), with Tukey HSD test demonstrating the case rate in July to be significantly greater than all other months.

Across 11 facilities, we identified 33 hepatitis A disease outbreaks (Figure 3C); 158 possible outbreak-related cases were diagnosed from April 2019 to September 2023. The mean duration of a hepatitis A outbreak was 1.2 months, varying from 1 month (multiple outbreaks) to 2 months (multiple outbreaks). The mean hepatitis A outbreak case count was 4.8 cases, varying from 3 cases (multiple outbreaks) to 24 cases (Krome North SPC in July 2021). The number of outbreaks per facility was partially skewed, ranging from 1 outbreak (multiple facilities) to 9 outbreaks (Krome North SPC) across facilities. Most cases were reported outside of outbreaks (328 of 486 cases [67.5%]).

Hepatitis A geospatial clustering analyses visually suggested case rates were highest across facilities in Texas, Louisiana, and Arizona, although no clusters were identified (global Moran *I* = −0.51; *P* = .69) (Figure 4C). Local Moran *I* analyses demonstrated no regional hepatitis A hot spots over the study period or within each calendar year. Additional results are shown in eAppendix 2 in Supplement 1.

## Discussion

In this case series, the epidemiologic patterns of 3 vaccine-preventable infectious diseases—influenza, mumps, and hepatitis A—within ICE detention facilities managed by IHSC underscore the value of vaccinating staff and detained individuals. Seasonal influenza and hepatitis A vaccines along with routine MMR (measles, mumps and rubella) vaccines may blunt the spread of infections diagnosed in ICE facilities. Although case counts and rates varied, some facilities (eg, Krome North SPC, Miami, Florida) had elevated case rates across diseases. Expected seasonal patterns were observed for influenza (December peak) and hepatitis A (July peak). Although outbreaks were most notable for influenza, mumps cases were clustered in a 1-year multifacility outbreak, and hepatitis A cases continue to increase. Although case rates for all diseases were highest in Southern facilities (Texas, Louisiana, Arizona, and Florida), these infections affected migrants nationwide without clear geospatial trends. Expanded vaccine-preventable disease campaigns alongside decarceration, decongestion, and facility ventilation and hygiene efforts can protect the health of migrants and staff, and further national biosecurity.

Case counts were lowest early in the study, likely reflecting underreporting in evolving documentation procedures. Although facility case rates varied significantly, a few facilities consistently reported the highest rates across diseases. We found that case rates across all 3 infections were elevated throughout 2021, overlapping with the transition to pandemic protocols. Influenza has shown nearly year-round transmission within the ICE system with a postpandemic increase. Although influenza case rates remained elevated, rates in many facilities were lower than national estimates while following similar seasonal trends. Despite potential differences in diagnostic and surveillance practices between ICE facilities and the general population, the relatively low influenza rate in facilities managed by IHSC highlights either success with transmission control or underreporting. We propose that the operations of positive deviance facilities with lower rates serve as a case study for facilities with high case rates, if underreporting is not the cause.

Mumps epidemiology demonstrated a 1-year, multifacility clustering in 2019 accounting for over two-thirds of reported cases (177 of 252 cases [70.2%]). The hepatitis A case rate has increased across facilities, primarily since the pandemic start with most cases reported outside of outbreaks (328 of 486 cases [67.5%]). High variation in disease transmission may reflect true trends; to further evaluate this, quality control and measurement of disease surveillance efforts are needed.^3,31^

A principal goal in exploring seasonal variations was to inform vaccination strategies to protect individuals in detention, staff, and families.^32^ From 2019 to 2023, there was significant seasonal variation in influenza (December peak) and hepatitis A (July peak) burden, approximating national trends.^33^ IHSC practitioners may anticipate continuing surges, and consider universal MMR and hepatitis A vaccine administration and seasonal influenza vaccination programs. Serial testing protocols during high-transmission periods, as undertaken during the pandemic, may aid in the prevention of contagious airborne diseases, including influenza.

Geospatial patterns suggested that Southern facilities, particularly in Texas and Louisiana, reported the highest case rates of all 3 diseases. Additionally, high case rates were found in Arizona facilities for influenza and Florida facilities for hepatitis A. We cannot differentiate whether geospatial differences are attributable to variations in regional management, diagnostic practices, characteristics of persons detained, and/or local transmission patterns. Overall, transmission within IHSC-managed facilities nationally had no significant autocorrelation. We urge increased transparency of case data for all staff—direct employees, contractors, and contingent workers—to improve disease transmission insights and further tailor prevention efforts. Our data, alongside previous analyses of migrant congregation settings, support campaigns specifically for staff to mitigate the bidirectional transmission risk among and between facility workers and detained migrants.^34^ Collection of baseline vaccination coverage may support resource prioritization.

Outbreak patterns varied across the diseases studied. Most outbreaks (79 of 128 outbreaks [61.7%]) were due to influenza. On average, influenza outbreaks were longest in duration and largest in case burden, compared with mumps and hepatitis A outbreaks, and occurred throughout the study period. Moreover, most influenza cases reported occurred within outbreaks (1739 of 2035 cases [85.5%]). Mumps cases were clustered over 1 year (2019) in shorter, multifacility outbreaks affecting the fewest facilities (8 of 20 facilities [40.0%]). Although hepatitis A outbreaks were shortest in duration and lowest in case burden, outbreak frequency has continued increasing. As we did not consider case clusters in proximity but across different months (eg, a case on February 28 and 2 cases on March 1), there may be additional outbreaks not measured.

This study reaffirms findings of infectious burden within ICE facilities, with additional insights on facility-level variation and post–COVID-19 pandemic trends. The burden of preventable infections among migrants accompanies evidence of increased case burdens in recent years, with clear seasonal surges in vaccine-preventable influenza and hepatitis A.^1,2^ Additionally, we found case demographics (30-35 years old, predominately male individuals) mirrored those of the detained migrant population.^35^ Although the infection risk was shared across detained migrants of all countries of origin, individuals from select countries of origin contributed larger case proportions possibly due to the facilities they were sent to, underlying risk, or migration routes. Although most analyses to date have been performed at the system level (across all ICE facilities), our work demonstrates high variation in risk (estimated by case rate) across facilities. We saw high, year-round influenza case rates, as seen previously, which may be driven by the detention of individuals from diverse regions with year-round influenza transmission and exposure to varying strains.^1,3,36,37^ Influenza case rates in facilities were overwhelmingly lower than those across the general US population excluding unique facility seasonal peaks. Our finding of a high risk of hepatitis A confirms findings in other global migrant populations.^38^ Given the risk to migrants and US residents alike, our findings support increased data transparency and expanded vaccination within ICE facilities.^2,31^

Individuals in ICE detention centers are particularly vulnerable to respiratory and enteric infections due to a convergence of risk factors. Many detained individuals have undertaken long journeys in crowded and poorly ventilated conditions, and those residing in the US prior to detention may have experienced housing instability or overcrowded living environments. Additionally, vaccination coverage in countries of origin may have been suboptimal. Detention centers often exhibit high levels of crowding, unhygienic conditions, and inadequate ventilation, further exacerbating risk. Alongside environmental interventions and vaccinations, reducing unnecessary detention through decarceration efforts can reduce risks.

### Limitations

We have limitations in our data and their interpretation. First, our results only reflect trends in 20 facilities staffed by IHSC, with the majority of the 147 facilities (as of 2024) operated by contractors and local governments without data available. Second, despite the extended mean length of detention (33.3 days) suggesting many cases were facility-acquired, documentation limits distinguishing from community-acquired cases. Additionally, we lack insight on diagnostic methods that may affect false-positive and false-negative rates. We lack data on vaccination receipt or coverage within facilities. Finally, without facility-level monthly population estimates, our case rates are not accurate point-estimates and serve as annualized-trend estimates.

## Conclusions

In this case series study, we found that vaccine-preventable disease risk has increased in ICE detention facilities over time, with high variability across facilities, suggesting the potential quality-improvement benefits of reviewing compliance with IHSC mandates. Trends in vaccine-preventable disease case rates follow a rapidly growing daily detention population during this study period that outpaced moderate annual budget increases. Funding for vaccine-preventable infection programs will support the critical work of IHSC to protect the health of detained migrants, ICE employees, and the broader US population.

## Notes

Supplement 1. eAppendix 1. Technical appendix eAppendix 2. Supplemental results eFigure 1. Three month sliding average of monthly case count over time from 2013 through 2023 eFigure 2. Three month sliding average of monthly case count over time stratified by reporting detention facility from 2013 through 2023 eFigure 3. Three month sliding average of case rate (per 100,000 person-months) over time by reporting detention facility from 2019 through 2023 with all facility labels included eFigure 4. Three month sliding average of case rate (per 100,000 person-months) over time by reporting detention facility from 2019 through 2023 with labels for facilities with highest case rates eFigure 5. Three month sliding average of monthly diagnosed cases over time from 2019 through 2023 eFigure 6. Three month sliding average of monthly case counts over time stratified by reporting detention facility from 2019 through 2023 eFigure 7. Three month sliding average of monthly influenza case count across ICE system and nationally over time from 2019 through 2023 eFigure 8. Three month sliding average of monthly influenza case rate (per 100,000 person-days) across ICE system and nationally over time from 2019 through 2023 eTable. Demographic, policy compliance and detention statistics of reporting facilities

Supplement 2. Data Sharing Statement
