# Supplementary material for: Epidemiology of 3 Vaccine-Preventable Infectious Diseases Within US Immigration Detention Centers
Source: JAMA Netw Open. 2025 Oct 22;8(10):e2544278. doi: 10.1001/jamanetworkopen.2025.44278 (PMC12547590; doi:10.1001/jamanetworkopen.2025.44278)
Supplement: Supplement 1. — eAppendix 1. Technical appendix eAppendix 2. Supplemental results eFigure 1. Three month sliding average of monthly case count over time from 2013 through 2023 eFigure 2. Three month sliding average of monthly case count over time stratified by reporting detention facility from 2013 through 2023 eFigure 3. Three month sliding average of case rate (per 100,000 person-months) over time by reporting detention facility from 2019 through 2023 with all facility labels included eFigure 4. Three month sliding average of case rate (per 100,000 person-months) over time by reporting detention facility from 2019 through 2023 with labels for facilities with highest case rates eFigure 5. Three month sliding average of monthly diagnosed cases over time from 2019 through 2023 eFigure 6. Three month sliding average of monthly case counts over time stratified by reporting detention facility from 2019 through 2023 eFigure 7. Three month sliding average of monthly influenza case count across ICE system and nationally over time from 2019 through 2023 eFigure 8. Three month sliding average of monthly influenza case rate (per 100,000 person-days) across ICE system and nationally over time from 2019 through 2023 eTable. Demographic, policy compliance and detention statistics of reporting facilities [file jamanetwopen-e2544278-s001.pdf]

Supplemental Online Content

Gupta R, Winslow D, Gupta R, Vermund SH. Epidemiology of 3 vaccine-preventable infectious diseases within US immigration detention centers. *JAMA Netw Open*. 2025;8(10):e2544278. doi:10.1001/jamanetworkopen.2025.44278

eAppendix 1. Technical appendix
eAppendix 2. Supplemental results
eFigure 1. Three month sliding average of monthly case count over time from 2013 through 2023
eFigure 2. Three month sliding average of monthly case count over time stratified by reporting detention facility from 2013 through 2023
eFigure 3. Three month sliding average of case rate (per 100,000 person-months) over time by reporting detention facility from 2019 through 2023 with all facility labels included
eFigure 4. Three month sliding average of case rate (per 100,000 person-months) over time by reporting detention facility from 2019 through 2023 with labels for facilities with highest case rates
eFigure 5. Three month sliding average of monthly diagnosed cases over time from 2019 through 2023
eFigure 6. Three month sliding average of monthly case counts over time stratified by reporting detention facility from 2019 through 2023
eFigure 7. Three month sliding average of monthly influenza case count across ICE system and nationally over time from 2019 through 2023
eFigure 8. Three month sliding average of monthly influenza case rate (per 100,000 person-days) across ICE system and nationally over time from 2019 through 2023
eTable. Demographic, policy compliance and detention statistics of reporting facilities

This supplemental material has been provided by the authors to give readers additional information about their work.

© 2025 Gupta R et al. *JAMA Network Open*.

eAppendix 1. Technical appendix

In this section, we provide additional methodology details regarding how the case rates were estimated and for various analyses.

Estimating case rate

In order to estimate the monthly case rate of each disease within a detention facility, we required case count and annualized estimates of the average daily population data per detention facility. Case rates were calculated for a subset of all facilities (N=18 of 20) due to limitations in access to population data.

Equation 1 was used to calculate the facility-level monthly case rate (per 100,000 person-months) of each infectious disease (influenza, mumps and hepatitis A). We assumed 30 days per month.

$$[1] \quad case\ rate_{m,d,f} = \frac{100{,}000 * N_{m,d,f}}{ADP_{y,f} * 30}$$

$where,$

$case\ rate$	= Case rate of disease per 100,000 person-months
N	= Total cases
ADP	= Average daily population (reported annually)
m	= Month
d	= Disease (influenza, mumps or hepatitis A)
f	= Detention facility
y	= Year

Equation 2 was used to calculate the national monthly case rate (per 100,000 person-months) of influenza.

$$[2] \quad National\ case\ rate_{m} = \frac{100{,}000 * N_{m}}{330{,}000{,}000}$$

$where,$

$National\ case\ rate$	= National case rate of influenza per 100,000 person-months
N	= Total cases of influenza nationally
m	= Month

All analyses were programmed in R version 3.6.2 (R Foundation for Statistical Computing; Vienna, Austria) and conducted in Stata/IC version 15.1 (StataCorp LP; College Station, United States), with the files and instructions for use available online (see main text reference to Github repository).

© 2025 Gupta R et al. *JAMA Network Open*.

eAppendix 2. Supplemental results

In this section, we provide a summary of the epidemiological trends of influenza, mumps and hepatitis A case counts across reporting facilities (N=20) within U.S. Immigration and Customs Enforcement (ICE) for the extended study period of November 2013 through October 2023. In addition, we repeat the outbreak analysis for each disease during the extended study period. These results support the primary analysis of trends in case rate and case count from January 2019 through October 2023.

Overview

From November 2013 through October 2023, there were 2,137 reported cases of Influenza, 521 reported cases of Hepatitis A, 263 reported cases of Mumps, 24 reported cases of Measles, 2 reported cases of Typhoid, and 1 reported case of Tetanus.

Trend analysis

From November 2013 through October 2023, the number of Influenza cases diagnosed per month across the system generally increased over time with an average of 17.8 cases diagnosed monthly, ranging from 0 cases (multiple months) to 276 cases (December 2021) (eFigure 3). At the facility level, the average number of Influenza cases diagnosed per month was 0.9 cases and varied across facilities from 0.1 cases (Varick Street SPC) to 3.1 cases (Montgomery Processing Center) diagnosed monthly (eFigure 4).

The number of Mumps cases diagnosed per month across the system, from November 2013 through October 2023, varied over time with an average of 2.2 cases diagnosed monthly, ranging from 0 cases (multiple months) to 61 cases (June 2019) (eFigure 3). At the facility level, the average number of Mumps cases diagnosed per month was 0.1 cases and varied across facilities from 0 cases (multiple facilities) to 0.5 cases (multiple facilities) diagnosed monthly (eFigure 4).

The number of Hepatitis A cases diagnosed per month across the system, from November 2013 through October 2023, generally increased over time with an average of 4.3 cases diagnosed monthly, ranging from 0 cases (multiple months) to 40 cases (July 2021) (eFigure 3). At the facility level, the average number of Hepatitis A cases diagnosed per month was 0.2 cases and varied across facilities from <0.1 cases (multiple facilities) to 0.9 cases (Krome North SPC) diagnosed monthly (eFigure 4).

We find notably fewer reported cases of all three infections prior to 2018. This finding may be due to a combination of factors including changes in ICE prevention protocols, increased use of detention facilities in recent years, increased lengths of stay, and changes in reporting protocols and data storage practices.

Outbreak analysis

From November 2013 through October 2023, 82 Influenza outbreaks (eFigure 1) were identified across 15 facilities. Across the system, 1774 Influenza outbreak cases were diagnosed with outbreaks found from January 2018 to October 2023. On average, the mean duration of an Influenza outbreak was 2.4 months, varying from 1 month (multiple outbreaks) to 13 months (September 2022 through September 2023 in South Texas ICE Processing Center). On average,

© 2025 Gupta R et al. *JAMA Network Open*.

the mean case count of an Influenza outbreak was 20.6 cases, varying from 3 cases (multiple outbreaks) to 185 cases (Port Isabel SPC from October 2021 through March 2022).

During the extended study period, 17 Mumps outbreaks (eFigure 1) were identified across 8 facilities. Across the system, 180 Mumps outbreak cases were diagnosed with outbreaks found from November 2018 to December 2019. On average, the mean duration of a Mumps outbreak was 1.7 months, varying from 1 month (multiple outbreaks) to 6 months (May 2019 through October 2019 in Port Isabel SPC). On average, the mean case count of a Mumps outbreak was 10.6 cases, varying from 3 cases (multiple outbreaks) to 52 cases (Krome North SPC from May 2019 through July 2019).

No additional Hepatitis A outbreaks were identified in the extended study period compared to the primary analysis from January 2019 through October 2023. In total, 33 Hepatitis A disease outbreaks (eFigure 1) were identified across 11 facilities. Across the system, 158 Hepatitis A outbreak cases were diagnosed with outbreaks found from April 2019 to September 2023. On average, the mean duration of a Hepatitis A outbreak was 1.2 months, varying from 1 month (multiple outbreaks) to 2 months (multiple outbreaks). On average, the mean case count of a Hepatitis A outbreak was 4.8 cases, varying from 3 cases (multiple outbreaks) to 24 cases (Krome North SPC in July 2021).

© 2025 Gupta R et al. *JAMA Network Open*.

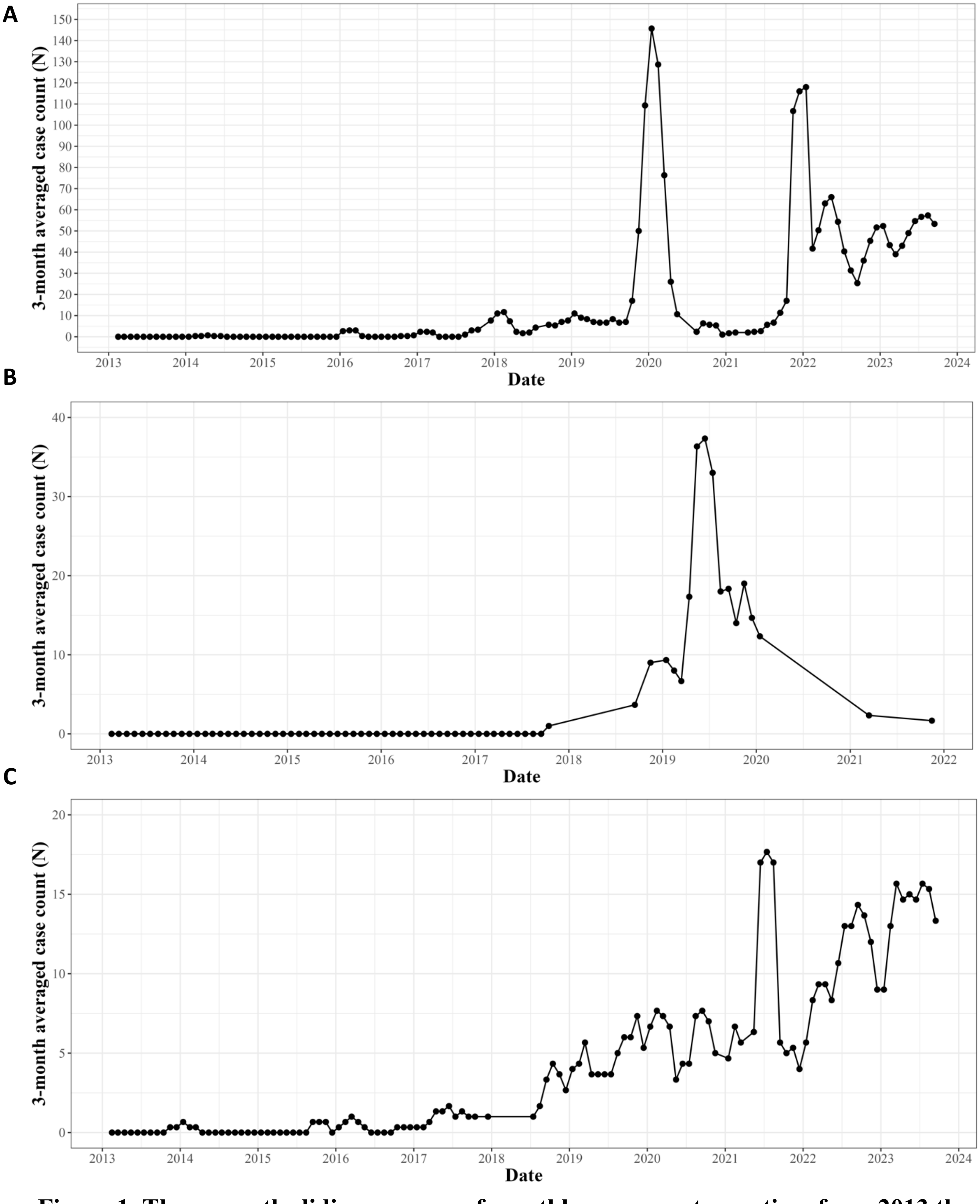

eFigure 1. Three month sliding average of monthly case count over time from 2013 through 2023. Panel A. Cases of influenza; Panel B. Cases of mumps; Panel C. Cases of hepatitis A. Note: Variation in Y-axis scale dependent on disease panel.

© 2025 Gupta R et al. *JAMA Network Open*.

A

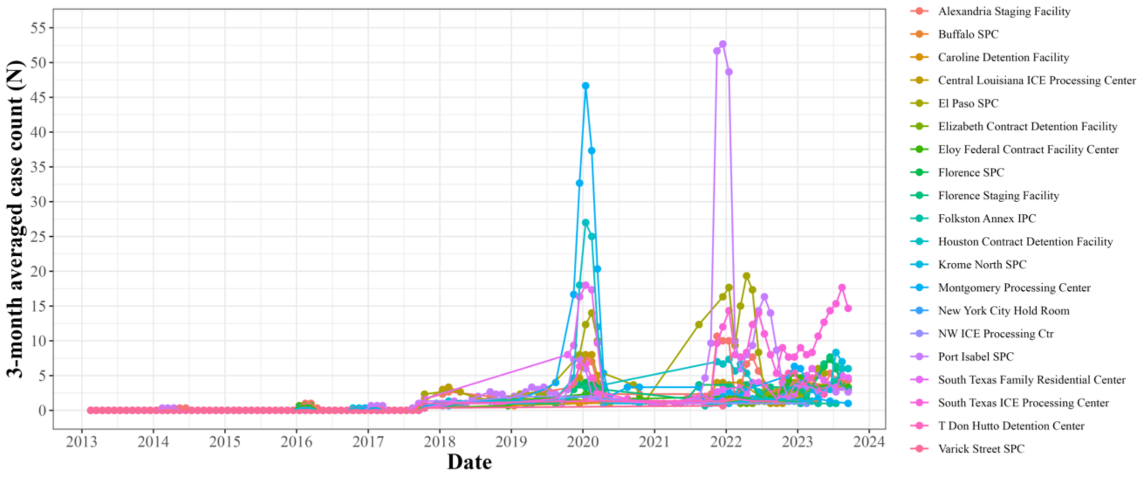

B

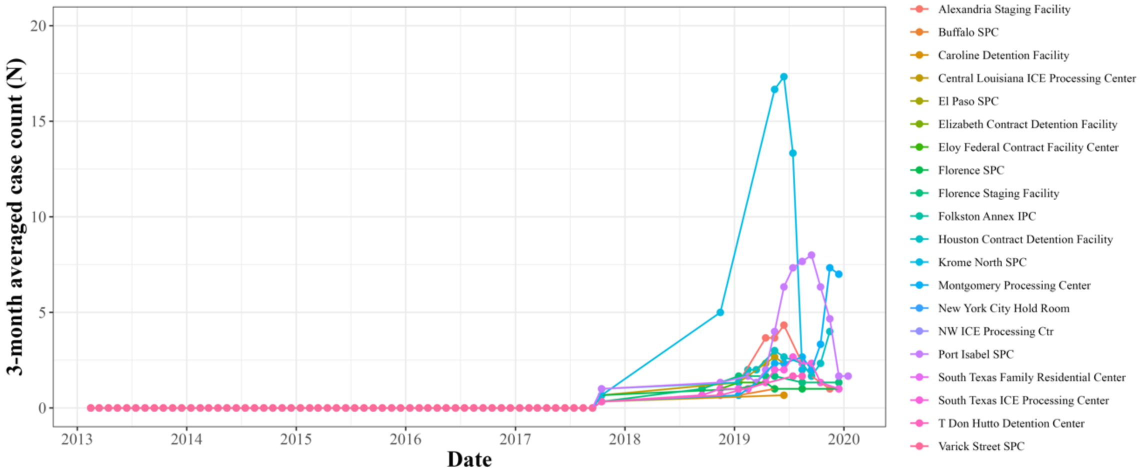

C

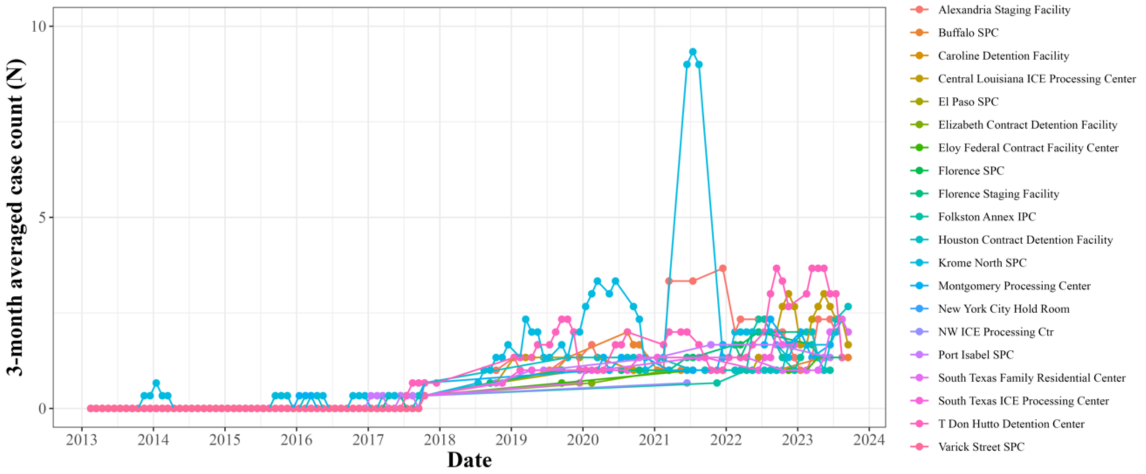

eFigure 2. Three month sliding average of monthly case counts over time stratified by reporting detention facility from 2013 through 2023. Panel A. Cases of influenza; Panel B. Cases of mumps; Panel C. Cases of hepatitis A. Note: Variation in Y-axis scale dependent on disease panel. Any facility with no cases reported in a month is given a 0 during the study period the trend lines are overlayed in reverse alphabetical order (e.g., all facilities reported 0 mumps cases throughout 2014).

© 2025 Gupta R et al. *JAMA Network Open*.

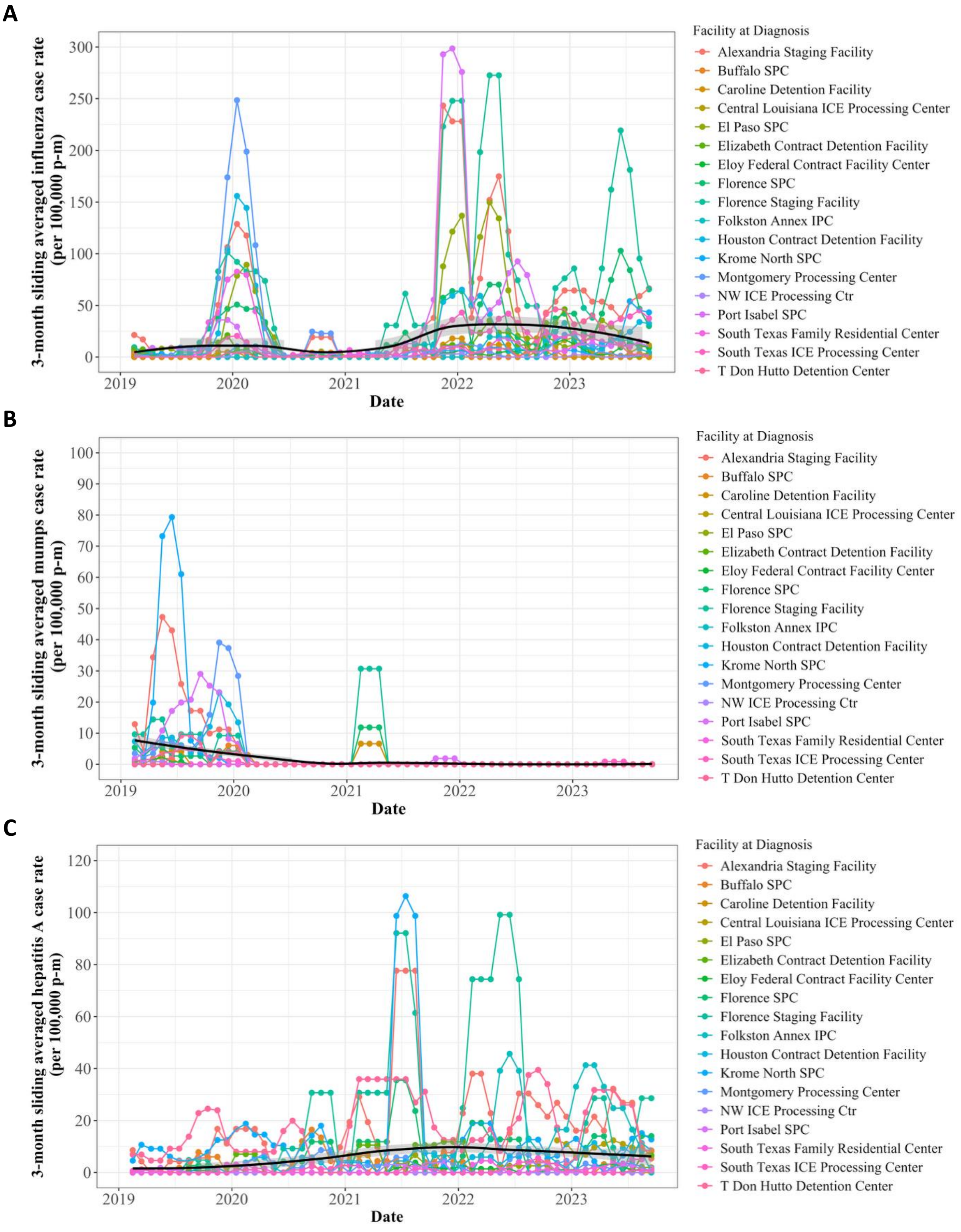

© 2025 Gupta R et al. *JAMA Network Open*.

D

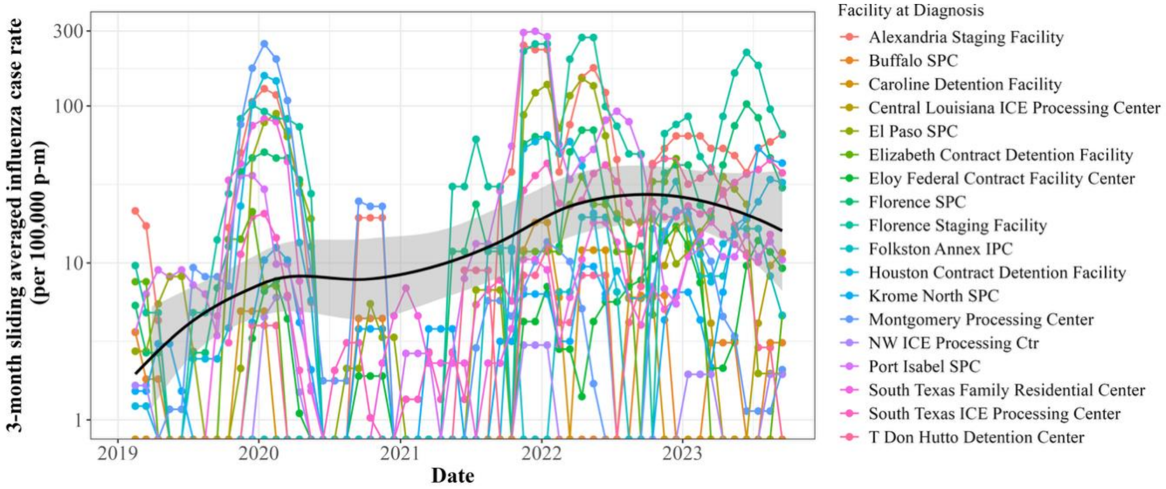

E

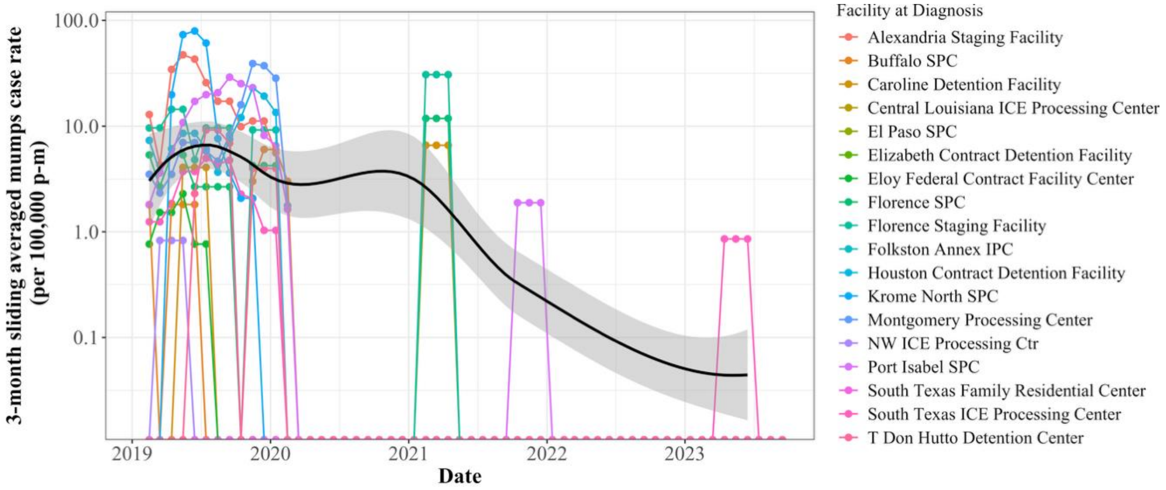

F

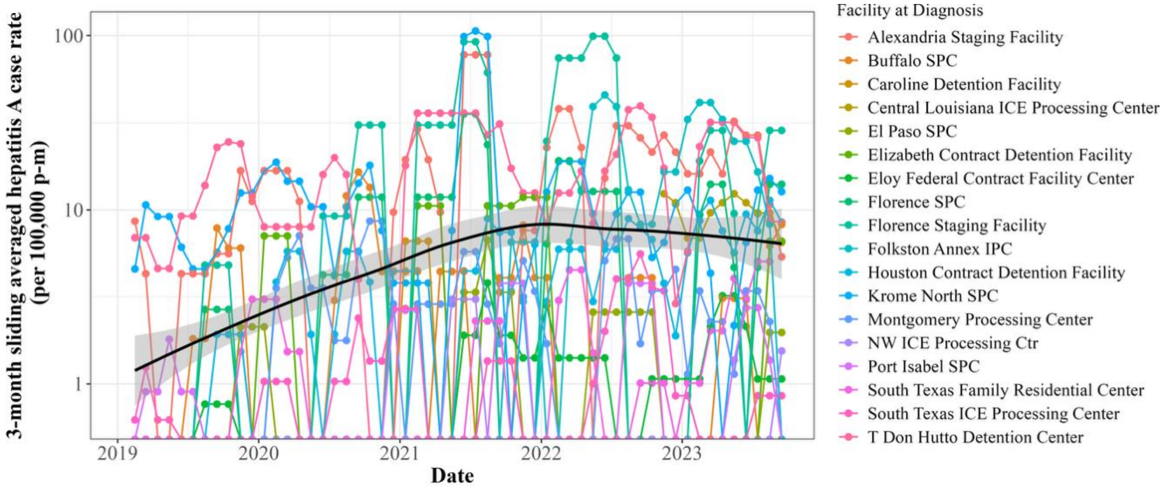

eFigure 3. Three month sliding average of case rate (per 100,000 person-months) over time stratified by reporting detention facility from 2019 through 2023. Panel A. Case rate of influenza on linear scale; Panel B. Case rate of mumps on linear scale; Panel C. Case rate of hepatitis A on linear scale; Panel D. Case rate of influenza on log scale; Panel E. Case rate of mumps on log scale; Panel F. Case rate of hepatitis A on log scale. Black line is the mean, unweighted facility-level case rate with a shadow of the standard deviation. Note: Variation in Y-axis scale dependent on disease panel.

© 2025 Gupta R et al. *JAMA Network Open*.

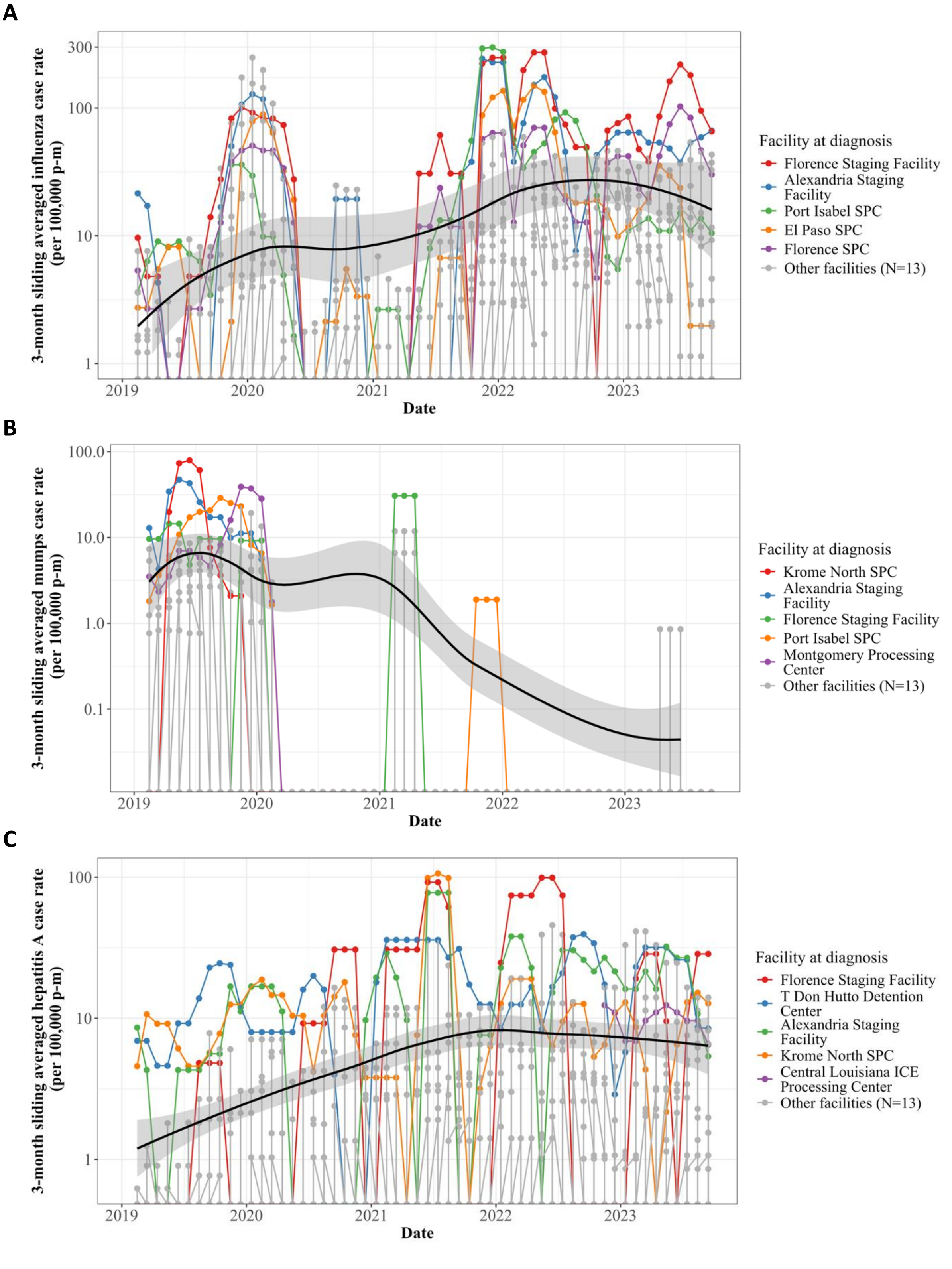

eFigure 4. Three month sliding average of case rate (per 100,000 person-months) over time by reporting detention facility from 2019 through 2023 with labels for facilities with highest case rates. For each disease, we provide distinct labels and line colors for the five facilities with the

© 2025 Gupta R et al. *JAMA Network Open*.

highest average disease-specific case rate from 2019 through 2023. All other reporting facilities are colored gray and labeled as “Other facilities.” A complete list of all facilities is available in Supplemental eFigure 1. Panel A. Case rate of influenza on log scale; Panel B. Case rate of mumps on log scale; Panel C. Case rate of hepatitis A on log scale. Black line is the mean, unweighted facility-level case rate with a shadow of the standard deviation. Note: Variation in Y-axis scale dependent on disease panel.

© 2025 Gupta R et al. *JAMA Network Open*.

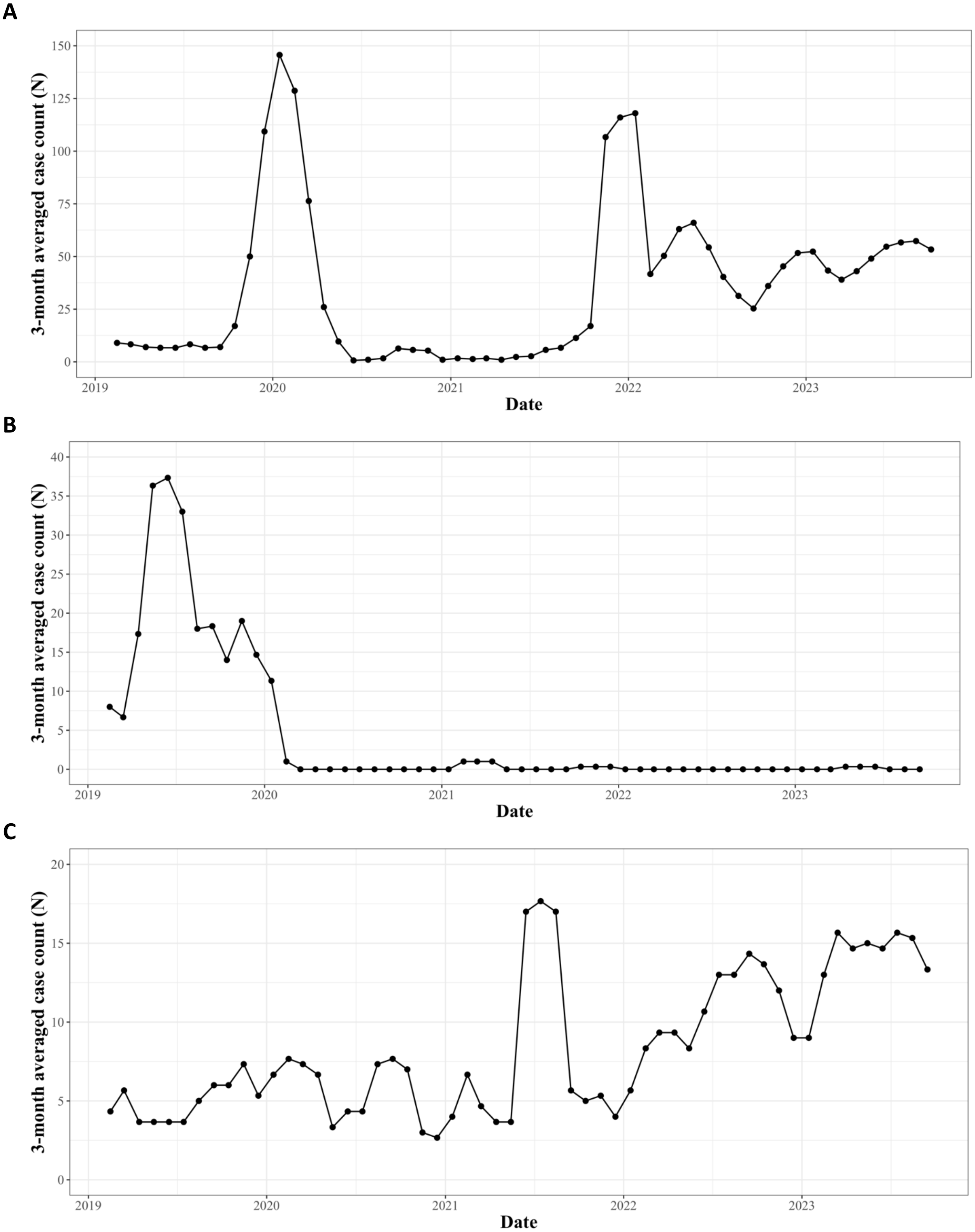

eFigure 5. Three month sliding average of monthly diagnosed cases over time from 2019 through 2023. Panel A. Cases of influenza; Panel B. Cases of mumps; Panel C. Cases of hepatitis A. Note: Variation in Y-axis scale dependent on disease panel.

© 2025 Gupta R et al. *JAMA Network Open*.

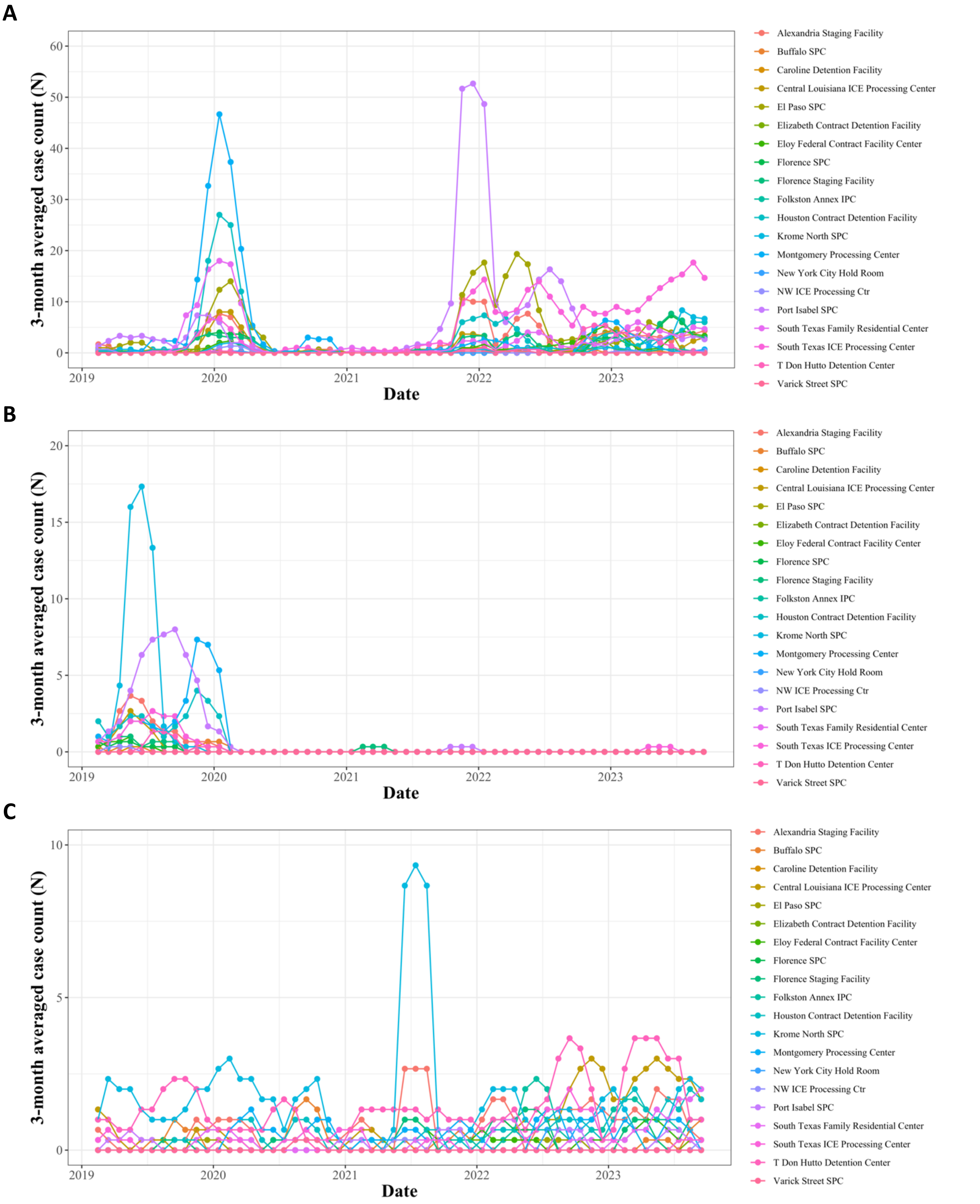

eFigure 6. Three month sliding average of monthly case counts over time stratified by reporting detention facility from 2019 through 2023. Panel A. Cases of influenza; Panel B. ases of mumps; Panel C. Cases of hepatitis A. Note: Variation in Y-axis scale dependent on disease panel.

© 2025 Gupta R et al. *JAMA Network Open*.

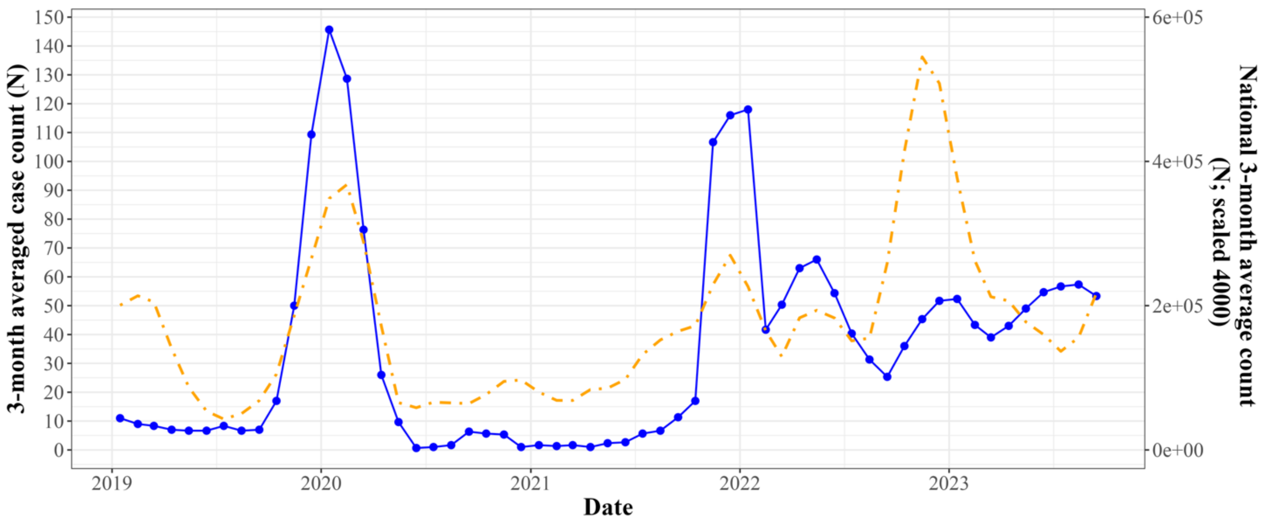

eFigure 7. Three month sliding average of monthly influenza case counts across ICE system and nationally over time from 2019 through 2023. Note: Orange dashed line is 3-month sliding average influenza case count across the United States. Blue solid line is 3-month sliding average influenza case count across reporting ICE facilities. Note difference in scales of national and ICE influenza case counts.

© 2025 Gupta R et al. *JAMA Network Open*.

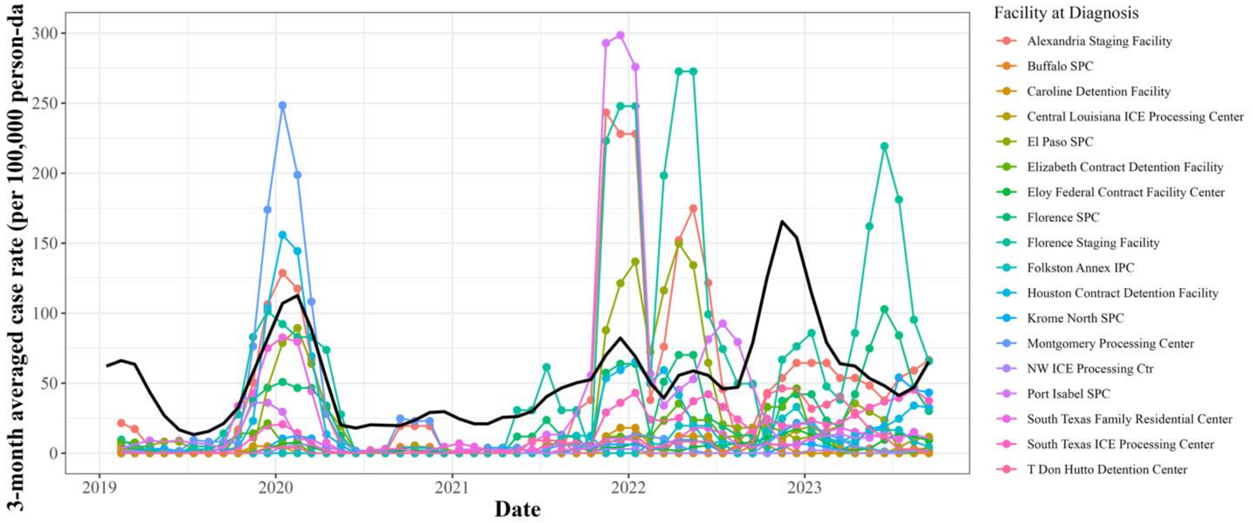

eFigure 8. Three month sliding average of monthly influenza case rate (per 100,000 person-days) across ICE system and nationally over time from 2019 through 2023. Black line is 3-month sliding average influenza case rate across the United States.

© 2025 Gupta R et al. *JAMA Network Open*.

eTable. Demographic, policy compliance and detention statistics of reporting facilities

Facility	Location	Male/Female	Average Length of Stay (Days)					
			FY19	FY20	FY21	FY22	FY23	FY24
Alexandria Staging Facility	Alexandria, LA	Male	3	3	4	4	3	4
Buffalo SPC	Batavia, NY	Male/Female	73	99	116	68	61	57
Caroline Detention Facility	Bowling Green, VA	Male/Female	32	58	47	79	47	58
Central Louisiana ICE Processing Center	Jena, LA	Male/Female	n.a.[1]	n.a.[1]	n.a.[1]	n.a.[1]	39	42
El Paso SPC	El Paso, TX	Male/Female	10	15	22	21	38	37
Elizabeth Contract Detention Facility	Elizabeth, NJ	Male/Female	56	46	31	13	17	17
Eloy Federal Contract Facility Center	Eloy, AZ	Male/Female	40	84	29	18	22	31
Florence SPC	Florence, AZ	Male	9	13	5	4	7	11
Florence Staging Facility	Florence, AZ	Male	2	2	2	1	2	3
Folkston Annex IPC	Folkston, GA	Male	35	38	41	32	26	40
Houston Contract Detention Facility	Houston, TX	Male/Female	35	40	28	27	30	33
Krome North SPC	Miami, FL	Male	24	28	33	46	44	38
Montgomery Processing Center	Conroe, TX	Male/Female	19	29	26	29	33	39
NW ICE Processing Ctr	Tacoma, WA	Male/Female	74	91	65	55	66	73
Port Isabel SPC	Los Fresnos, TX	Male/Female	7	16	7	7	10	11
South Texas Family Residential Center	Dilley, TX	Male/Female	12	44	12	12	28	n.a.[1]
South Texas ICE Processing Center	Pearsall, TX	Male/Female	30	72	49	28	40	42
T Don Hutto Detention Center	Taylor, TX	Female (2019-2021); Male (2022-2024)	28	90	21	17	34	52
Varick Street SPC	n.a.[1]	n.a.[1]	n.a.[1]	n.a.[1]	n.a.[1]	n.a.[1]	n.a.[1]	n.a.[1]

© 2025 Gupta R et al. *JAMA Network Open*.

New York City Holding Room	n.a.[1]	n.a.[1]	n.a.[1]	n.a.[1]	n.a.[1]	n.a.[1]	n.a.[1]	n.a.[1]

[1]No information available in annual ICE detention statistic report
[2]Last Inspection Standard collected from FY2024 ICE detention statistic report for all facilities, except for South Texas Family Residential Center where it was collected from FY2023 report.
[3]PBNDS denotes Performance-based national detention standards.
[3]FRS denotes Family residential standards.

© 2025 Gupta R et al. *JAMA Network Open*.

eTable. Demographic, policy compliance and detention statistics of reporting facilities (continue)

Facility	Last Inspection Standard (as of 2024)[2,3,4]	Average Daily Population (ADP)					
		FY19	FY20	FY21	FY22	FY23	FY24
Alexandria Staging Facility	2013 Errata PBNDS	259	199	115	146	207	298
Buffalo SPC	2016 Revised PBNDS	611	369	251	273	359	549
Caroline Detention Facility	2016 Revised PBNDS	273	226	168	184	201	241
Central Louisiana ICE Processing Center	2016 Revised PBNDS	n.a.[1]	n.a.[1]	n.a.[1]	n.a.[1]	808	1116
El Paso SPC	2016 Revised PBNDS	813	522	331	430	563	761
Elizabeth Contract Detention Facility	2016 Revised PBNDS	293	157	105	94	168	239
Eloy Federal Contract Facility Center	2016 Revised PBNDS	145	1010	584	789	1041	1418
Florence SPC	2016 Revised PBNDS	415	262	94	174	238	387
Florence Staging Facility	2016 Revised PBNDS	231	121	36	45	117	202
Folkston Annex IPC	2016 Revised PBNDS	n.a.[1]	194	89	170	135	241
Houston Contract Detention Facility	2016 Revised PBNDS	908	577	249	374	588	786
Krome North SPC	2016 Revised PBNDS	728	533	293	351	513	583
Montgomery Processing Center	2016 Revised PBNDS	951	626	387	653	976	1170
NW ICE Processing Ctr	2016 Revised PBNDS	1342	739	363	371	570	719
Port Isabel SPC	2016 Revised PBNDS	1231	678	420	588	812	976

© 2025 Gupta R et al. *JAMA Network Open*.

South Texas Family Residential Center	FRS	1075	726	484	738	1099	n.a.[1]
South Texas ICE Processing Center	2016 Revised PBNDS	1788	1076	822	1109	1298	1588
T Don Hutto Detention Center	2016 Revised PBNDS	482	278	124	266	385	417
Varick Street SPC	n.a.[1]	n.a.[1]	n.a.[1]	n.a.[1]	n.a.[1]	n.a.[1]	n.a.[1]
New York City Holding Room	n.a.[1]	n.a.[1]	n.a.[1]	n.a.[1]	n.a.[1]	n.a.[1]	n.a.[1]

[1]No information available in annual ICE detention statistic report
[2]Last Inspection Standard collected from FY2024 ICE detention statistic report for all facilities, except for South Texas Family Residential Center where it was collected from FY2023 report.
[3]PBNDS denotes Performance-based national detention standards.
[4]FRS denotes Family residential standards.

© 2025 Gupta R et al. *JAMA Network Open*.
