# Supplementary material for: Epidemiology of 3 Vaccine-Preventable Infectious Diseases Within US Immigration Detention Centers
Source: JAMA Netw Open. 2025 Oct 22;8(10):e2544278. doi: 10.1001/jamanetworkopen.2025.44278 (PMC12547590; doi:10.1001/jamanetworkopen.2025.44278)
Supplement: Supplement 2. — Data Sharing Statement [file jamanetwopen-e2544278-s002.pdf]

Data Sharing Statement

Gupta. Epidemiology of 3 Vaccine-Preventable Infectious Diseases Within US Immigration Detention Centers. *JAMA Netw Open*. Published October 22, 2025.
doi:10.1001/jamanetworkopen.2025.44278

Data
Data available: Yes
Data types: Deidentified participant data, Data dictionary
How to access data: De-identified data and data dictionaries will be made available upon emailed requests to ribhav.gupta97@gmail.com
When available: With publication

Supporting Documents
Document types: Statistical/analytic code
How to access documents: https://github.com/rgupta97/ICE_ID
When available: With publication

Additional Information
Who can access the data: De-identified data will be provided to anyone requesting the data with clear intention for use of the data.
Types of analyses: The data will be provided for any research purposes.
Mechanisms of data availability: Data will be made available with investigator support.
Any additional restrictions: None.
